# Nitrogen removal via the nitrite pathway during wastewater co-treatment with ammonia-rich landfill leachates in a sequencing batch reactor

**DOI:** 10.1007/s11356-014-2641-1

**Published:** 2014-02-27

**Authors:** S. Fudala-Ksiazek, A. Luczkiewicz, K. Fitobor, K. Olanczuk-Neyman

**Affiliations:** 1 Sanitary Engineering, Faculty of Civil and Environmental Engineering, Gdansk University of Technology, Gabriela Narutowicza Str. 11/12, 80-233 Gdansk, Poland; 2 Department of Water and Wastewater Technology, Faculty of Civil and Environmental Engineering, Gdansk University of Technology, Gabriela Narutowicza Str. 11/12, 80-233 Gdansk, Poland

**Keywords:** Landfill leachates, Wastewater, Co-treatment, SBR, Partial nitrification (PN), Ammonia-oxidising bacteria (AOB), Nitrite-oxidising bacteria (NOB)

## Abstract

The biological treatment of ammonia-rich landfill leachates due to an inadequate C to N ratio requires expensive supplementation of carbon from an external carbon source. In an effort to reduce treatment costs, the objective of the study was to determine the feasibility of nitrogen removal via the nitrite pathway during landfill leachate co-treatment with municipal wastewater. Initially, the laboratory-scale sequencing batch reactor (SBR) was inoculated with nitrifying activated sludge and fed only raw municipal wastewater (RWW) during a start-up period of 9 weeks. Then, in the co-treatment period, consisting of the next 17 weeks, the system was fed a mixture of RWW and an increasing quantity of landfill leachates (from 1 to 10 % by volume). The results indicate that landfill leachate addition of up to 10 % (by volume) influenced the effluent quality, except for BOD_5_. During the experiment, a positive correlation (*r* ^2^ = 0.908) between ammonia load in the influent and nitrite in the effluent was observed, suggesting that the second step of nitrification was partially inhibited. The partial nitrification (PN) was also confirmed by fluorescence in situ hybridisation (FISH) analysis of nitrifying bacteria. Nitrogen removal via the nitrite pathway was observed when the oxygen concentration ranged from 0.5 to 1.5 mg O_2_/dm^3^ and free ammonia (FA) ranged from 2.01 to 35.86 mg N-NH_3_/dm^3^ in the aerobic phase. Increasing ammonia load in wastewater influent was also correlated with an increasing amount of total nitrogen (TN) in the effluent, which suggested insufficient amounts of assimilable organic carbon to complete denitrification. Because nitrogen removal via the nitrite pathway is beneficial for carbon-limited and highly ammonia-loaded mixtures, obtaining PN can lead to a reduction in the external carbon source needed to support denitrification.

## Introduction

**Fig. 1:**
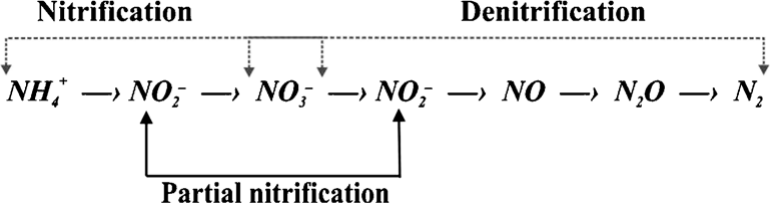
Partial nitrification–denitrification through nitrites (adapted from Ruiz et al. 2006)

Landfill leachates are generated at landfill sites as a result of precipitation, infiltration, compaction and waste degradation. The quality and quantity of landfill leachates are highly variable and are affected by many factors, including the amount of precipitation, weather conditions, waste type and decomposition rate (Renou et al. 2008). Thus, even today, suitable treatment of leachates is regarded as a worldwide problem. Considering leachate characteristics, technical possibilities, regulatory requirements and cost effectiveness, multistage treatment systems (e.g. physical, chemical and biological processes) are often proposed. Except for components of concern, such as heavy metals and xenobiotic organic compounds (Slack et al. 2005; Christensen et al. 2001), landfill leachates contain high age-dependent concentrations of organic matter and ammonia. The biological treatment of landfill leachates is generally considered effective if the biodegradable fraction of organic matter is adequate to complete denitrification (TN to BOD ratio <2). Young landfill leachates (during the acid phase) contain relatively high amounts of organic carbon available for microorganisms; however, the increase in ammonia is accompanied by an increase in the non-biodegradable fraction of organic matter over time (during the methanogenic phase followed by the aerobic phase) (Kulikowska and Klimiuk 2008; Zhang et al. 2007; Lema et al. 1988). Due to an inadequate COD to TN ratio, the biological treatment of landfill leachate often requires external carbon sources to support denitrification. The expense of such supplementation is not economically feasible; therefore, in recent years, new biological approaches in landfill leachate treatment (partial nitrification (PN) process via nitrite, SHARON, and Anammox) have become the subject of numerous laboratory- and pilot-scale studies (Blackburne et al. 2008; Liu et al. 2010; Sri Shalini and Joseph 2012; Van der Star et al. 2007). PN (nitritation without nitratation) is tested (Fig. 1) because less oxygen and assimilable carbon are consumed due to the inhibition of the second step of nitrification. Compared with traditional nitrification/denitrification via nitrate, PN via nitrite reduces the aeration consumption in the nitrification stage by approximately 25 % and the organic matter needed for denitrification by approximately 40 % (Pollice et al. 2002; Turk and Mavinic 1989; Zhou et al. 2011). Simultaneously, profitable advantages, such as a higher denitrification rate and lower surplus sludge production, have also been noted (Guo et al. 2009; Aslan et al. 2009; Zhou et al. 2006; Ciudad et al. 2005). To achieve PN (shortcut nitrification), the activity of nitrite-oxidising bacteria (NOB) must be selectively reduced without affecting the activity of ammonia-oxidising bacteria (AOB).

Nevertheless, treatment of landfill leachates may be difficult due to the complex composition of leachates and the variability of flow rates. Thus, combined biological treatment of landfill leachates with municipal wastewater appears to have technological and economical advantages. It was estimated that landfill leachate generation in Poland varies from 12 to 22 % of the annual precipitation (lower and higher values are typical for younger and older landfills respectively). Thus, in the span of a year, the volumetric contribution of landfill leachates to wastewater in municipal wastewater treatment systems typically does not exceed 0.4 % (Quant et al. 2009). However, the ratio of landfill leachates to wastewater may increase during heavy rainfall. This study tested the efficiency of wastewater co-treatment with increasing additions of landfill leachates (1, 2, 5 and 10 % by volume) using a laboratory-scale sequencing batch reactor (SBR). During the SBR experiment, the effectiveness of co-treatment by analysing the chemical oxygen demand (COD), the biochemical oxygen demand over a 5-day period (BOD_5_) and total nitrogen (TN) and total phosphorus (TP) removal was tested. In addition, the laboratory-scale SBR was used to investigate the effect of increasing the ammonia concentration and low dissolved oxygen (DO) on nitrification and nitrite accumulation. To monitor the PN process, detailed periodical determinations of the ammonia utilisation rate (AUR), nitrite production rate (NPR), and nitrate production rate (NAPR) under aerobic conditions were employed for 5 and 10 % additions of landfill leachates to wastewater. Changes in specific bacteria responsible for the first and second steps of nitrification were also analysed by fluorescence in situ hybridisation (FISH).

## Materials and methods

### Materials

Activated sludge and raw municipal wastewater (RWW, after screening) were obtained from the Gdansk-Wschod wastewater treatment plant (WWTP), operating in a modified University of Cape Town (mUCT) system. This WWTP serves a population equivalent of 700,000 PE with daily flow of approximately Q_av._ = 96,000 m^3^/d. The WWTP also receives wastewater from local industries (6.5 %), including the food industry, shipbuilding and the chemical industry.

The landfill leachates originated from the municipal solid waste plant (MSWP) “Eko Dolina” in Lezyce, which has been operating since 2003 and serves 440,000 inhabitants. The total amount of waste currently generated is approximately 180,000 Mg/year (municipal waste comprises 130,000 Mg), of which 50 % is recycled. The volume of landfill leachates was estimated to be 30,000 m^3^/year (considering that the average rainfall in the studied area is 600 mm/year (Lorenc 2005)). The leachates are collected at the bottom of the landfill prism by a drainage system and then pumped to the pretreatment system. After the reverse osmosis process, treated landfill leachates are discharged into the municipal sewage system, and the concentrate is pumped back to the landfill prism.

### Experiment set-up

**Fig. 2:**
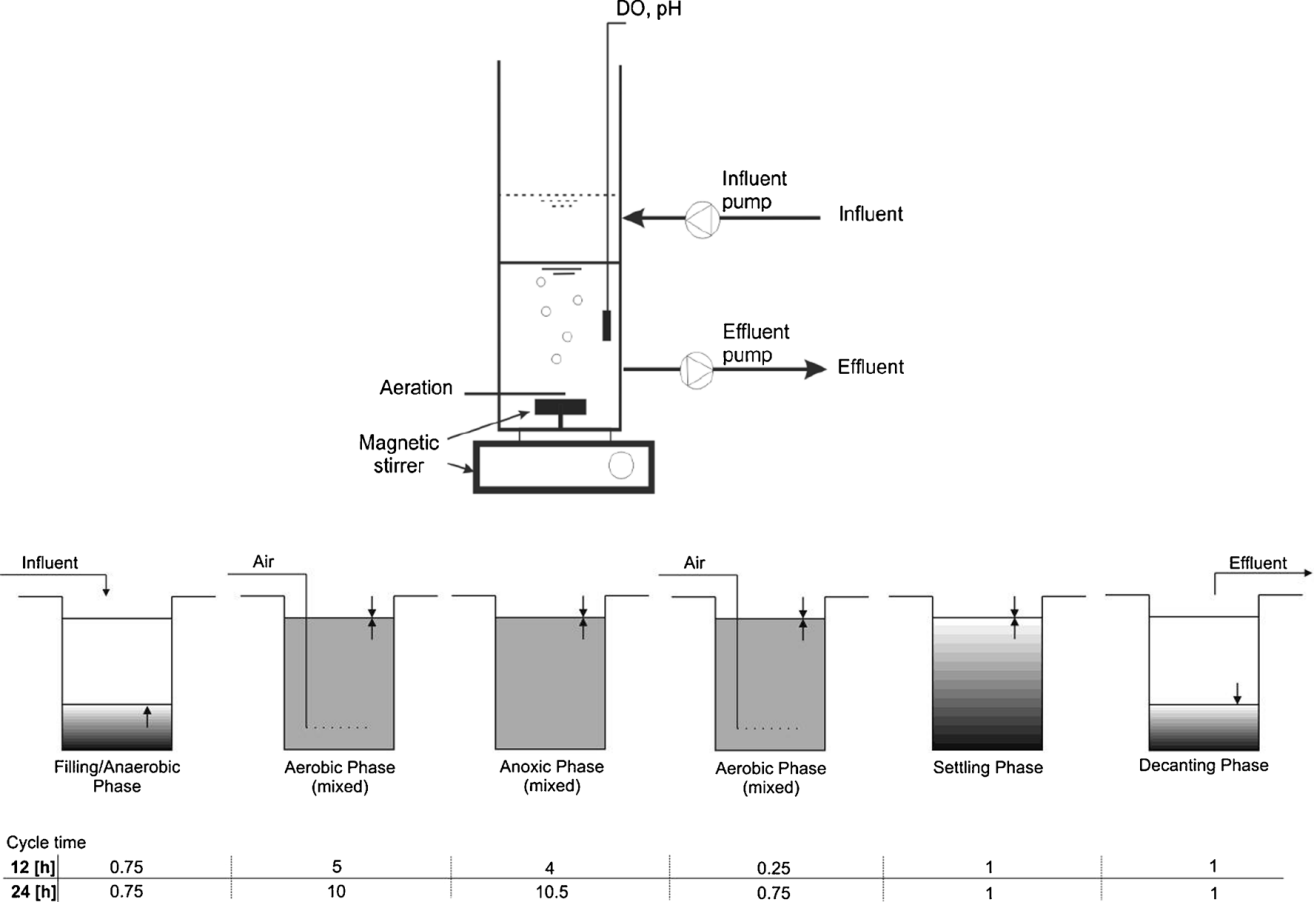
Schematic of the SBR with specified phases of the process

The laboratory-scale SBR was constructed as a cylindrical tank with a total volume of 8 dm^3^ (20 cm in diameter and 30 cm high) and equipped with a magnetic stirrer, an air micro-diffuser in the bottom, and online measurement of DO and pH (Fig. 2).

Initially, the SBR was inoculated with nitrifying activated sludge and was fed RWW during a 9-week start-up period. Over the next 17 weeks (the co-treatment period), a mixture of RWW with increasing quantities (from 1 to 10 % volume) of raw landfill leachates (RLL) was fed to the SBR. In this study, RWW was mixed with RLL at volumetric ratios of 1 % (RM1), 2 % (RM2), 5 % (RM5) and 10 % (RM10) and then supplied to the SBR system using a peristaltic pump. The SBR was operated in a thermostatic room at 20 ± 1 °C with a sludge retention time (SRT) of 70–92 days. The activated sludge concentration was 3.5 g dry mass/dm^3^. The concentration of DO in the aerobic phase was initially set to 1.0 ± 0.5 mg O_2_/dm^3^. The total cycle times for the SBRs were 12 h for 1 and 2 % addition of landfill leachates and 24 h for 5 and 10 %. Each SBR cycle consisted of six phases: anaerobic filling, aerobic reaction, anoxic reaction, aerobic reaction, settling and decantation (Fig. 2).

### Analytical methods

The activated sludge concentration, total and volatile suspended solid (TSS and VSS respectively), COD, BOD_5_ and concentrations of nitrite nitrogen (N-NO_2_), nitrate nitrogen (N-NO_3_), ammonia nitrogen (N-NH_4_), and TN, phosphorus phosphate (P-PO_4_), TP, chlorides (Cl^−^) and sulphates (SO_4_^2−^) were determined according to the Standard Methods (APHA 2005). The analyses were performed using duplicate samples. Additionally, free ammonia (FA) concentration and free nitrous acid (FNA) were estimated using the following equation, which was proposed by Anthonisen et al. (1976):

1 $$ \mathrm{FA}\kern0.3em \mathrm{as}\kern2.77695pt {\mathrm{NH}}_3\left(\mathrm{mg}\kern2.77695pt \mathrm{N}/{\mathrm{dm}}^3\right)=\frac{17}{14}\times \frac{{\mathrm{NH}}_4\left(\mathrm{mg}/{\mathrm{dm}}^3\right)\times {10}^{\mathrm{pH}}}{{\mathrm{K}}_{\mathrm{b}}/{\mathrm{K}}_{\mathrm{w}}+{10}^{\mathrm{pH}}} $$

where *K*_*b*_ to *K*_*w*_ ratio = *e* ^6344/273 + *T*^

2 $$ \mathrm{FNA}\kern2.77695pt \mathrm{as}\kern2.77695pt {\mathrm{HNO}}_2\left(\mathrm{mg}\kern2.77695pt \mathrm{N}/{\mathrm{dm}}^3\right)\frac{47}{14}\times \frac{N-N{O}_2\left( mg/d{m}^3\right)}{K_a\times {10}^{pH}} $$

where *K*_*a*_ = *e* ^(2300/273 + *T*)^

Several parameters were determined to analyse the PN process. During the nitrification phase, samples were taken from the SBR every 15–60 min and then immediately filtered by a Whatman GF/C filter (Whatman Ltd.) to separate the activated sludge from the treated mixture and analyse TN, N-NH_4_, N-NO_2_, and N-NO_3_. Then, the AUR, NPR, and NAPR were calculated under aerobic conditions. Five detailed PN analyses were carried out during the study, two for RM5 and three for RM10.

### FISH analyses of AOB and NOB

Because the accumulation of nitrite was observed in the SBR process, the samples of activated sludge treating RM5 and RM10 were withdrawn from the reactor at the end of the nitrification phase to test for the presence of AOB and NOB. As a reference sample, the initial activated sludge, obtained from the Gdansk-Wschod WWTP, was also analysed. All samples were fixed within 2 h of collection according to the protocol recommended by Nielsen et al. (2009) for gram-negative bacteria. Samples were stored at −20 °C until they were processed. Next, before hybridisation, refrigerated samples were gently homogenised, immobilised on glass slides and then dehydrated in increasing concentrations of ethanol (50, 90, and 98 %). In this study, common AOB (β-proteobacterial) and NOB (*Nitrobacter* and *Nitrospira*) were tested using commercially available oligonucleotide probes together with a EUB-mix probe (Table 1). A non-binding Non338 probe was used to indicate the non-specific fluorescence and act as the negative control. All probes were synthesised by Biomers.net, and probes of interest were labelled with fluorescein, whereas EUB-mix and Non338 probes were labelled with CY3. The hybridisation procedure was performed according to Nielsen et al. (2009). The relative abundance of probe-targeted bacteria was estimated by comparing an area fluorescing with group-specific probes with the area fluorescing with the EUB-mix probe and was evaluated by enumerating 30 randomly chosen microscopic fields. Images were taken using a Nikon 80i epifluorescence microscope (with total magnification × 1,200). This system consists of an EpiF microscope accessory kit, a high-definition colour CCD camera, and a PC with image acquisition and image analysis software (Nis-Elements, Japan).

**Table 1:** 16S rRNA oligonucleotide probes used to identify the AOB and NOB bacteria (Nielsen et al. 2009)

Probe	Target	Sequence (5′–3′)	F%
EUB_mix_ (EUB338 I–III)	Most bacteria, *Planctomycetales* and *Verrucomicrobialea*	GCT GCC TCC CGT AGG AGT GCA GCC ACC CGT AGG TGT GCT GCC ACC CGT AGG TGT	35 or 40
Non338	control (nonsense probe)	CGACGGAGGGCATCCTCA 2	20
Ammonia-oxidising bacteria (AOB )			
Nso1225	β-proteobacterial AOB	CGC CAT TGT ATT ACG TGT GA	35
Nitrite-oxidising bacteria (NOB)			
Nit3	*Nitrobacter*	CCT GTG CTC CAT GCT CCG	40
		Competitor: CCT-GTG-CTC-CAG-GCT-CCG	
Ntspa 662	*Nitrospira*	GGA ATT CCG CGC TCC TCT	35
		Competitor: GGA ATT CCG CTC TCC TCT	

*F* formamide

### Statistical analysis

For the concentrations and removal efficiency of TN, N-NH_4_, COD, BOD and TSS, the standard uncertainty was calculated using the assumption of rectangular distribution. The reported uncertainty was an expanded uncertainty calculated using a coverage factor of *k* = 1.65, which gives a confidence level of approximately 95 %. For AUR, NUR, and NAPR, however, the standard uncertainty was calculated using the assumption of Gaussian distribution. The reported uncertainty was an expanded uncertainty calculated using a coverage factor of *k* = 2, which gives a confidence level of approximately 95 %. Median and standard deviation were used to indicate the central tendency and spread of data.

In this study, the chi-square test of independence (χ^2^; Devore 2012) was employed to calculate the relationship between two variables, assuming that there is no association between the removal efficiency and concentration of pollutant in RWW and RM1, RM2, RM5, and RM10. The null hypothesis (H_0_), in the used χ^2^ test, assumes that the above variables are independent. Differences were considered statistically significant if *p* < α = 0.05. Statistical analyses were performed using the MS Excel 2007 and Origin Pro 9.0 software.

## Results

### Characteristics of the RLL and influent mixtures (RM1, RM2, RM5 and RM10)

The quality of RLL varied widely during this study (Table 2), particularly N-NH_4_ concentration, which ranged from 1,395 to 3,040 mg N-NH_4_/dm^3^. The average COD to BOD_5_ ratio, the above six, indirectly indicated that the leachates were rich in low and non-biodegradable COD and, together with the high TN to BOD_5_ ratio (on average 4.67), confirmed the medium age of the studied landfill site (operated since 2003). Additionally, the leachates were found to contain relatively high chloride concentrations (ranging from 2,354.08 to 3,828.92 mg Cl^−^/dm^3^, 2,822 mg Cl^−^/dm^3^ on average). Although chloride is commonly found in landfill leachate, the high chloride concentration at the studied landfill site is likely the result of the municipal landfill plant operation system (concentrate after reverse osmosis is returned back to the landfill prism).

**Table 2:** Characteristic parameters of the SBR influents

Probe	Parameter (mg/dm^3^)								
		TN	N-NH_4_	TP	BOD	COD	TSS	TN/BOD	COD/BOD
RLL	μ ± U	2,045 ± 0.5	1,895 ± 0.5	16.3 ± 0.05	485.3 ± 0.05	2,766 ± 0.5	56.1 ± 0.165	4.67 ± 0.002	6.17 ± 0.003
	σ	489	527	2.1	141.3	528	16.2	2.17	2.25
RWW	μ ± U	74.5 ± 0.05	48.6 ± 0.05	9.9 ± 0.05	442.1 ± 0.05	544 ± 0.5	295.8 ± 0.165	0.17 ± 0.0003	1.28 ± 0.0001
	σ	11.9	12.0	3.0	83.2	152	65.3	0.03	0.03
RM1^a^	μ ± U	113.5 ± 0.05	96.6 ± 0.05	12.3 ± 0.05	622.5 ± 0.05	816 ± 0.5	312.0 ± 0.165	0.18 ± 0.0002	1.31 ± 0.0004
	σ	14.0	12.8	1.9	57.9	61	87.2	0.02	0.02
RM2^b^	μ ± U	141.7 ± 0.05	127.9 ± 0.05	11.8 ± 0.05	606.9 ± 0.05	892 ± 0.5	405.1 ± 0.165	0.24 ± 0.0002	1.49 ± 0.001
	σ	10.0	13.2	1.6	95.7	97	184.4	0.05	0.02
RM5^c^	μ ± U	234.2 ± 0.05	219.1 ± 0.05	11.1 ± 0.05	508.3 ± 0.05	885 ± 0.5	346.8 ± 0.165	0.45 ± 0.0003	1.79 ± 0.0005
	σ	35.5	34.6	3.2	142.6	139	210.3	0.06	0.08
RM10^d^	μ ± U	274.4 ± 0.05	254.9 ± 0.05	10.6 ± 0.05	500.0 ± 0.05	880 ± 0.5	352.7 ± 0.165	0.56 ± 0.0003	1.78 ± 0.0006
	Σ	16.7	16.0	1.6	88.0	120	170.2	0.10	0.03

Mean (μ) ± standard uncertainty (U), level of confidence is 95 %

σ standard deviation, *RLL* raw landfill leachates, *RWW* raw wastewater, *TN* total nitrogen, *N-NH*_*4*_ ammonia nitrogen, *TP* total phosphorus, *BOD* biochemical oxygen demand, *COD* chemical oxygen demand, *TSS* suspended solid

^a–d^RWW with the increasing volumetric addition of RLL (RM11 %, RM2 2 %, RM5 5 %, RM10 10 %)

Because RWW with increasing volumetric addition of RLL (from 1 to 10 %) was biologically treated in this study, the influent mixtures (RM1, RM2, RM5 and RM10) were characterised by biodegradability and denitrification feasibility, expressed as the COD to BOD_5_ and TN to BOD_5_ ratios respectively. In both cases, as the addition of landfill increases from RM1 to RM10, the ratio gradually increases, with a COD to BOD_5_ ratio from 1.31 to 1.78 and a TN to BOD_5_ ratio from 0.18 to 0.56. This result indicated that conditions were favourable for microbial degradation (COD to BOD_5_ ratio <2) throughout the experiment, although the amount of carbon in RM2, RM5 and RM10 was insufficient to complete denitrification (TN to BOD_5_ ratio >0.2). Certain impacts to the nitrification process were expected because the TN in the treated mixtures (RM1, RM2, RM5 and RM10) increased due to the high-ammonia load introduced to the RWW with landfill leachates (from 48.6 mg N-NH_4_/dm^3^ for RWW to 254.9 mg N-NH_4_/dm^3^ for RM10; Table 2).

### Effectiveness of treatment in the SBR

**Fig. 3:**
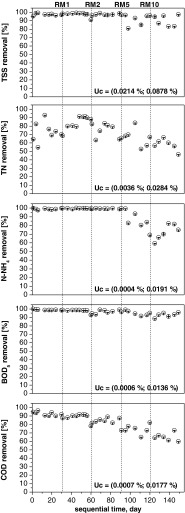
Removal of the main wastewater components in the co-treatment process. The *dotted lines* indicate the addition of landfill leachates. The reported uncertainty (U) is an expanded uncertainty calculated using a coverage factor of *k* = 1.65, which gives a confidence level of approximately 95 %

**Fig. 4:**
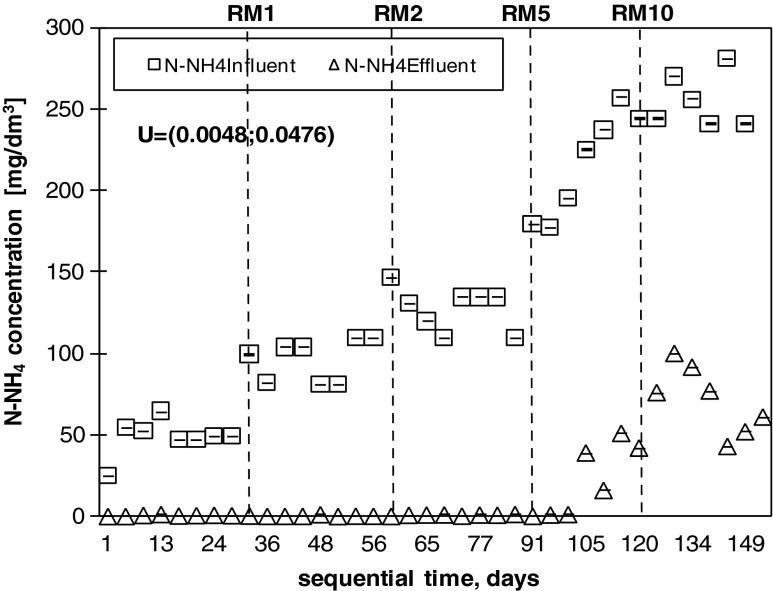
Removal of ammonia nitrogen in the SBR. The *dotted lines* indicate the addition of landfill leachates. The reported uncertainty (U) is an expanded uncertainty calculated using a coverage factor of *k* = 1.65, which gives a confidence level of approximately 95 %.

In the stable portion of the start-up period, high-efficiency removal (above 90 %) was obtained for BOD_5_, COD and TSS (Fig. 3). Highly effective N-NH_4_ reduction (up to 96 %) was also noted (Fig. 4), whereas TN removal varied between 55 and 93 % (73 % on average; Fig. 3). An average reduction of 80 % was achieved for TP.

**Fig. 5:**
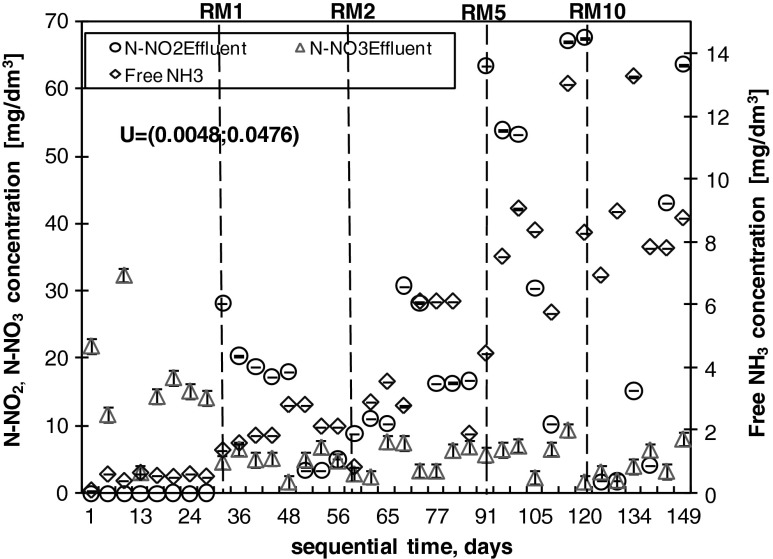
Effect of free ammonia, nitrite and nitrate to the background concentration of ammonia nitrogen in the SBR effluent. The *dotted lines* indicate the addition of landfill leachates. The reported uncertainty (U) is an expanded uncertainty calculated using a coverage factor of *k* = 1.65, which gives a confidence level of approximately 95 %

During the co-treatment period, the increasing addition of landfill leachates from 1 to 10 % (volume) influenced the effluent quality metrics, with the exception of BOD_5_. Although the landfill leachates reached 5 and 10 % (volume) of the influent mixture (RM5 and RM10 respectively), the effectiveness of BOD_5_ removal remained above 90 % (Fig. 3), whereas the amount of COD removed decreased from 90 to approximately 60 % (Fig. 3). The efficiency of TSS removal also decreased from 98 % for RM1 to 91 % for RM5 and RM10. More than 80 % of phosphorus was removed at the end of the start-up period, whereas this removal amount gradually decreased in the co-treatment period from 56 % for RM1 to 40 % for RM10 (Fig. 3). The average TN reduction was 81, 80, 66 and 59 % for RM1, RM2, RM5 and RM10 respectively (Fig. 3), whereas the removal efficiency of ammonia was higher, with an average of 99.91 and 72 % for RM1 and RM2, RM5 and RM10 respectively (Figs. 3 and 4). During the start-up period, nitrate represented the majority (up to 100 %) of the TN in the effluent. In contrast, during the co-treatment of RWW with the addition of RLL, the average amount of nitrite (N-NO_2_) was 62 % for RM1, 66 % for RM2, 61 % for RM5 and 22 % for RM10 (Fig. 5). Nitrates (N-NO_3_) in the effluent decreased with the addition of landfill leachates and constituted an average of 31, 19, 8 and 4 % for RM1, RM2, RM5 and RM10 respectively. Detailed analyses of nitrogen utilisation were undertaken for RM5 and RM10 to clarify the cause of nitrite accumulation.

In this study chi-square test of independence was used to evaluate the relationship between the removal efficiency and concentration of pollutants in RWW and RM1-10. According to the obtained results, the calculated value (χ^2^) is higher than the cricital value (χ^2^_α, df_) at* p < α = 0.05* and specific degree of freedom (d_f_) (Table 3); thus, we reject H_0_. The removal efficiency is therefore dependent on the concentration of pollutants.

**Table 3:** The results of the chi-squared test of independence (χ^2^)

Parameter	TSS	TN	N-NH_4_	BOD_5_	COD
α	0.05	0.05	0.05	0.05	0.05
*Df*	4	20	16	20	16
χ^2^	9.7	34.1	37.8	37.5	53.9
χ^2^_α; df_	9.5	31.4	26.3	31.4	26.3
results χ^2^ test	χ^2^ ≥ χ^2^_α; *df*_	χ^2^ ≥ χ^2^_α; *df*_	χ^2^ ≥ χ^2^_α; *df*_	χ^2^ ≥ χ^2^_α; *df*_	χ^2^ ≥ χ^2^_α; *df*_

*TSS* suspended solid, *TN* total nitrogen, *N-NH*_*4*_ ammonia nitrogen, *BOD* biochemical oxygen demand for 5-day period, *COD* chemical oxygen demand

### Conditions favouring PN

During the study, increasing amounts of nitrite were observed in the final effluent, suggesting PN and/or incomplete denitrification. To explain this phenomenon, a detailed analysis of nitrogen removal was undertaken for RM5 and RM10.

During the filling phase (45 min), the expected increase of TN was noted in the SBR, mainly as ammonia. Nitrite was also observed in the treated mixture; nitrite likely remained in the activated sludge after the previous cycle (as a result of low denitrification efficiency). This assumption was confirmed by nitrite utilisation in the anaerobic conditions of the filling phase, supported by the simultaneous increase of the amount of biodegradable carbon introduced to the SBR reactor with the influent mixture. Then, during the aerobic phase, the concentration of N-NH_4_ decreased, with an average AUR of 6.65 g N/(kg VSS · h) for RM5 and 7.90 g N/(kg VSS · h) for RM10 (Table 4). The average NPR measured in aerobic conditions was 5.61 g N-NO_2_/(kg VSS · h) and 7.05 g N-NO_2_/(kg VSS · h) during PN for RM5 and RM10 respectively, whereas the NAPR was comparatively low, with average values of 0.39 and 0.57 g N-NO_3_/(kg VSS · h) during PN for RM5 and RM10 (Table 4) respectively. The pH was above 8 during RM5 and RM10 (8.29–8.65 for RM5 and 8.31–8.57 for RM10).

**Table 4:** The rates of biochemical processes during PN analyses

RLL addition (%)	Number of PN analysis	Process temperature (°C)	Rate of biochemical processes (g N/kg VSS · h)		
			AUR	NPR	NAPR
5 % (RM5)	1	20	6.47 ± 0.93^a^	5.40 ± 0.56	0.36 ± 0.08
	2	20	6.82 ± 0.94	5.81 ± 0.63	0.42 ± 0.16
10 % (RM10)	1	20	7.11 ± 0.70	6.09 ± 0.99	0.62 ± 0.30
	2	20	6.96 ± 0.61	6.18 ± 0.64	0.50 ± 0.18
	3	20	9.64 ± 0.76	8.88 ± 1.17	0.57 ± 0.16

^a^ ± standard uncertainty (U), level of confidence 95 %

**Fig. 6:**
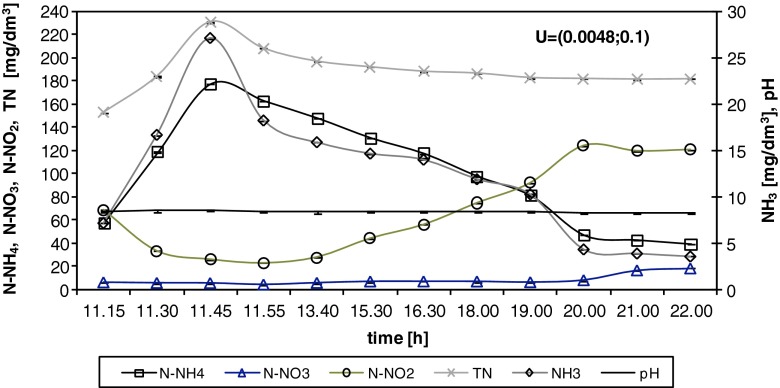
Example of the detailed analyses of PN in the SBR with 10 % addition of landfill leachates (RM10). The *vertical line* indicates the beginning of nitrification. The reported uncertainty (U) is an expanded uncertainty calculated using a coverage factor of *k* = 1.65, which gives a confidence level of approximately 95 %

Figure 6 presents the nitrogen utilisation during RM10 in detail. As mentioned above, nitrite was observed at the beginning of the filling phase (69 mg N-NO_2_/dm^3^), and its concentration gradually decreased over time. At the end of the filling phase, TN reached 231 mg N/dm^3^, with nitrite accounting for 11 % (26 mg N-NO_2_/dm^3^), nitrate for 2 % (26.3 mg N-NO_3_/dm^3^) and N-NH_4_ for 77 % (178.3 mg/dm^3^), calculated based on NH_3_, which reached 27.16 mg/dm^3^ (Fig. 6). Next, during the aerobic phase (10 h), the concentration of N-NH_4_ decreased from 178.3 to 39.8 mg N-NH_4_/dm^3^, with an AUR of 7.1 g N/(kg VSS · h) (Table 4). N-NO_2_ increased from 26.2 to 120.8 mg N-NO_2_/dm^3^ with an NPR of 6.1 g N/(kg VSS · h) (Table 4), whereas N-NO_3_ remained at a similar level throughout the nitrification process (Fig. 6). An increase in the nitrate concentration was only observed in the last 2 h of the aerobic phase, increasing from 8.1 to 18.5 mg N-NO_3_/dm^3^. After the denitrification process (10.5 h), 61 % of TN, including 43.1 mg N-NO_2_/dm^3^, 6.4 mg N-NO_3_/dm^3^, and 42 mg N-NH_4_/dm^3^, was removed (data not shown). The pH was above 8 (between 8.31 and 8.56) for the duration of the test (Fig. 6).

### FISH analyses

**Fig. 7:**
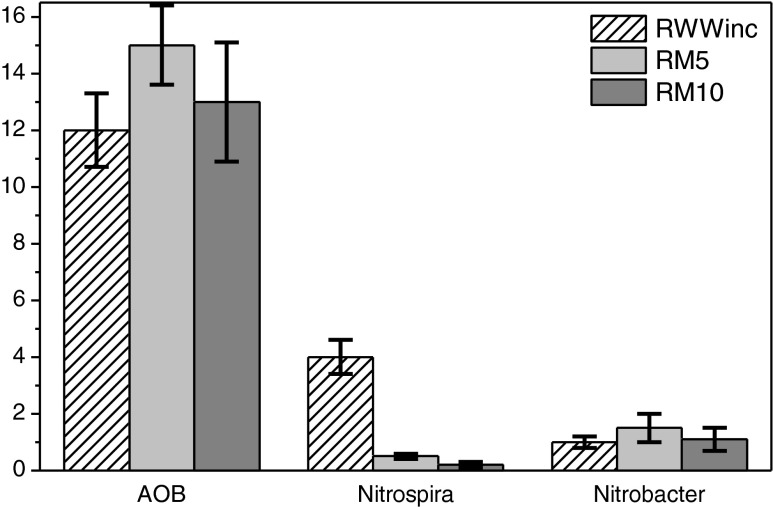
Relative abundance of AOB and NOB (*Nitrospira* and *Nitrobacter*) communities in RWWinc (activated sludge used for SBR inoculation) as well as in RM5 and RM10 (RWW with 5 and 10 % addition of landfill leachates)

The FISH analyses were carried out to determine the AOB and NOB communities during the accumulation of nitrite in the SBR. In the initial activated sludge, the relative abundance of β-proteobacterial AOB (targeted with the probe Nso1225) constituted approximately 12 ± 1.3 % of the total eubacterial population (targeted with the probe EUB-mix), whereas the NOB community (targeted with the probe Nit3 for *Nitrobacter* and Ntspa 662 for *Nitrospira*) accounted for approximately 5 ± 0.9 %. A predominance of *Nitrospira* was initially observed (Fig. 7); for RM5 and RM10, the relative abundance of *Nitrospira* did not exceed 0.5 %, whereas *Nitrobacter* accounted for up to 1.5 %, suggesting the inhibition of the second phase of nitrification. The addition of landfill leachates to the wastewater influenced the fractions of NOB and changed the morphological structure of the AOB and NOB microcolonies. In the initial activated sludge, both AOB and NOB communities were found to occur as typical spheroidical aggregates, composed of very closely packed cells. Together with the addition of landfill leachates (and simultaneous nitrite accumulation), the diameter of microcolonies decreased considerably, and the microcolonies were more randomly distributed in the flocks (especially NOB). Some microcolonies in the analysed activated sludge flocks were too small to observe compared with the background. Because RLL, and thus also RM1–RM10, contained large amounts of chloride, sulphate and other ions (data not shown), their contribution to the deterioration of NOB and AOB aggregates cannot be excluded.

## Discussion

### Biological co-treatment of landfill leachates in the SBR

Biological processes are frequently recommended for the removal of organic matter and nitrogen from landfill leachates (Peng et al. 2008, Sri Shalini and Joseph 2012; Yu et al. 2010). The biological co-treatment of wastewater and leachates appears to be a feasible technology. Yu et al. (2010) reported that the optimal ratio of landfill leachates to municipal wastewater volume in the anaerobic-anoxic-oxic (A2/O) bioreactor system is 1:500 (0.2 %). The authors' previous experiments revealed that in an SBR system, this value can range from 0.5 to 1 % (Fudala-Ksiazek et al. 2010). In this study, raw wastewater was co-treated in a laboratory-scale SBR with an increasing volumetric addition of landfill leachates from 1 to 10 % to establish the conditions favouring PN.

According to the results, even though the landfill leachates reached 10 % (volume) of the influent mixture, the effectiveness of BOD_5_ removal remained above 95 %, and TSS reduction efficiency achieved a level above 91 % (Fig. 3). The COD efficiency dropped from 90 % (RWW) to approximately 60 % (RM10) (Fig. 3) due to the increasing concentration of the non-biodegradable (inert) COD fraction to the total COD present in the influent mixtures (from 18 % for RWW to 30 % for RM10). The inert fraction of COD has been widely reported to increase with landfill site age (Bilgili et al. 2008; Kulikowska and Klimiuk 2008). Thus, during co-treatment, to avoid the problems connected with COD load above the discharge limit, the inert fraction of COD in the wastewater-landfill leachate mixture should not exceed 125 mg/dm^3^ or 75 % reduction of COD (Polish limits for WWTP effluents). In this study, the addition of up to 5 % landfill leachates meets the above assumption; however, in real technological systems, the addition of landfill leachates should be constantly monitored and corrected to obtain an acceptable level of COD in effluent due to the variable quality of landfill leachates.

The average reduction of TN in the SBR system was 81, 80, 66 and 59 % for RM1, RM2, RM5 and RM10 respectively (Fig. 3), whereas the average removal efficiency of ammonia was higher, with values of 99 % for RM1 and RM2, 91 % for RM5 and 72 % for RM10. The experiment was performed with almost no disturbances to the oxidation of ammonia for 1 and 2 % landfill leachate additions (Fig. 4). Although AOB are generally considered to be slowly growing chemolithoautotrophs and poor competitors for oxygen (Van Niel et al. 1993), the increasing ammonia concentration in the influent (from RWW to RM10) was followed by an increasing AUR, reaching 7.9 g N/(kg VSS · h) for RM10. Similarly, a high reduction of N-NH_4_ was also achieved by Yu et al. (2010) during the co-treatment of domestic wastewater with landfill leachates in a pilot-scale A2/O bioreactor. In this study, however, the efficient AUR was not followed by an efficient NPR, suggesting the inhibition of the second step of nitrification. This result was also confirmed by a detailed analysis of nitrogen compounds (TN, N-NH_4_, N-NO_2_ and N-NO_3_) appearing during the nitrification phase and by determination of AOB and NOB using FISH (as described in “Conditions favouring PN” and “FISH analyses”). The concentration of N-NO_3_ in the effluent decreased as N-NO_2_ started to appear in the SBR effluent (Fig. 5). The fluctuation of nitrite observed during the study has also been reported by others (Kim et al. 2006; Spagni and Marsili-Libelli 2009) and can be explained by the variation in the amount of FA (Fig. 5). The ammonia, nitrite and nitrate concentrations in the effluent suggested nitrogen removal via the nitrite pathway. The main advantages of PN over complete nitrification are associated with lower oxygen consumption during the nitrification process (up to 25 %), lower carbon requirements during denitrification (up to 40 %), and the reduced reactor volume due to lower hydraulic retention time (HRT) requirements, higher denitrification kinetics, and smaller sludge production (up to 300 %) (Pollice et al. 2002; Turk and Mavinic 1989; Zhou et al. 2011).

However, in this study, the efficiency of TN removal decreased from approximately 80 % for RM1 and RM2 to 66 % for RM5 and 59 % for RM10 (Fig. 3). Previous experiments have indicated that during co-treatment, special emphasis should be placed on the TN to BOD ratio (Bilgili et al. 2008; Spagni et al. 2008). To maintain high denitrification efficiency, the TN to BOD ratio should be lower than 0.2, however, it increased from 0.18 for RWW to 0.56 for RM10 with the addition of landfill leachates (Table 2). According to the obtained data, the internal assimilable organic carbon present in the treated mixtures (RM2–RM10) was not sufficient to complete denitrification, although the occurrence of the nitrite pathway in denitrification (after PN) could reduce the organic carbon required for denitrification by 35–40 % (Spagni and Marsili-Libelli 2009; Turk and Mavinic 1989). Thus, an external carbon source should be supplemented to maintain a high denitrification rate, as suggested by Spagni et al. (2008) and Wu et al. (2009). Increased nitrate/nitrite reduction efficiency by denitrification will increase the TN removal efficiency (Yu et al. 2010).

The presence of oxidised nitrogen compounds is also important for phosphorus utilisation by activated sludge. Conventional SBR systems are regarded as less effective and sensitive in terms of biological phosphorus uptake. Furthermore, the dephosphatation process displayed significant fluctuations (Fig. 3). The presence of nitrate and nitrite in the anaerobic phase may disturb the release of phosphate under anaerobic conditions. According to Zeng et al. (2011), nitrite levels below 10 mg N-NO_2_/dm^3^ did not inhibit P-uptake and release, whereas enhanced biological phosphorus removal (EBPR) was observed when the accumulation reached 20 mg N-NO_2_/dm^3^. Previous studies have indicated that in carbon-limited conditions, the main factor leading to EBPR deterioration could be competition with denitrifiers for carbon (Janssen et al. 2002; Quant et al. 2009).

### Factors influencing PN during the co-treatment period

In the start-up period, nitrate represented the major part (up to 100 %) of the TN in the final effluent (Fig. 5). In the co-treatment period, the nitrite contribution to the effluent gradually increased, suggesting the inhibition of the second phase of nitrification. Several factors, such as DO concentration, FA concentration, pH and temperature, influence ammonia and nitrite oxidation (Aslan et al. 2009; Ciudad et al. 2005; Zhang et al. 2011). The results obtained in this study confirmed that PN occurred during the wastewater co-treatment with landfill leachates (Figs. 5 and 6) and was connected with shifts in the AOB/NOB community structure.

*Nitrosospira* (AOB) and *Nitrospira* (NOB) are characterised by low growth rate, a high affinity for substrate growth, and the ability to survive at low substrate concentrations (k-strategists), whereas *Nitrosomonas* (AOB) and *Nitrobacter* (NOB) are able to grow rapidly at high substrate concentrations (r-strategists) (Manz et al. 1996; Shramm et al. 1998, 2000; Nogueria et al. 2002). Additionally, AOB possess a more versatile metabolism and higher affinity for oxygen (Schmidt et al. 2002; Schmidt et al. 2004) than NOB. In this study, the actual DO level was 1 ± 0.5 mg O_2_/dm^3^, and thus, the relatively low concentration could selectively favour some AOB, such as *Nitrosomonas europaea* (Park and Noguera 2004), and suppress the activity of NOB. In this study, the significant inhibition of tested nitrite oxidisers (particularly *Nitrospira*) was observed for RM5 and RM10; the relative abundance of NOB at RM10 did not exceed 1.5 % of the total eubacterial population, whereas the tested β-proteobacterial AOB constituted approximately 12 ± 1.3 % of the total eubacterial population. Enhanced ammonia oxidation can be supported by ammonia-oxidising archaea (AOA) (Park et al. 2006). Additionally, Schmidt (2009) demonstrated that under oxygen-depleted conditions, AOB used nitrogen dioxide as an electron acceptor instead of molecular oxygen and pyruvate or lactate instead of CO_2_ as a carbon source.

In the case of NOB, a DO concentration of 0.4–0.7 mg O_2_/dm^3^ was suggested as the limiting factor (Ma et al. 2009). In this study, the accumulation of nitrite (PN) occurred in the aerobic phase for DO concentrations of 0.5–1.5 mg O_2_/dm^3^ (data not shown) and ammonia concentrations above 80 mg N-NH_4_/dm^3^ (Fig. 4). Additionally, a positive correlation of nitrite with the increasing ammonia load in the influent was observed (r^2^ = 0.908), suggesting that inhibition of the second step of nitrification was also the result of FA being present in the treated mixture (Aslan et al. 2009; Kim et al. 2006). Although FA inhibits both steps of nitrification, it has a larger effect on NOB (Anthonisen et al. 1976). The first phase (ammonia oxidation) is inhibited at the level of 10–150 mg NH_3_/dm^3^, whereas the second phase (nitrite oxidation) is inhibited at the level of 0.1–1.0 mg/dm^3^ (Anthonisen et al. 1976).

With the continuous increase of N-NH_4_ (pH above 8), there was a constant increase in FA from 0.49 mg N-NH_3_/dm^3^ for RWW to 2.06, 3.78, 8.05 and 8.91 mg N-NH_3_/dm^3^ for RM1, RM2, RM5 and RM10 respectively (Fig. 5). Furthermore, NOB were inhibited. During the detailed analysis of the nitrification phase of RM5 and RM10, FA reached values between 2.01 and 35.86 mg N-NH_3_/dm^3^ (Fig. 6). The threshold FA concentration causing AOB and NOB inhibition found in the literature is different. According to Kim et al. (2006), FA completely inhibited AOB and NOB at a concentration of 78 mg N-NH_3_/dm^3^, and at FA concentrations between 14 and 17 mg N-NH_3_/dm^3^, only nitrite oxidation was selectively inhibited, whereas ammonium was oxidised to nitrite.

Zhang et al. (2011) studied the effect of pH on nitrite accumulation and the nitrifier community and found that there was no significant change in AOB and NOB activity across a pH range of 7.0–8.5; however, pH has a strong influence on FA concentration because it assigns the distribution of N-NH_4_/N-NH_3_ equilibrium. According to Zhang et al. (2010), pH is a critical parameter for monitoring PN and has a close relationship with the available substrate (N-NH_4_). Zhang et al. (2010) suggest that FA is the main inhibitor of nitrification under high pH conditions (pH >8), whereas FNA is the main inhibitor under low pH conditions (pH <7.5). Pambrun et al. (2006) and Zhang et al. (2011) reported that a pH above 7 has a significant influence on the increasing concentration of FA in the SBR reactor and causes inhibition of NOB, particularly under high N-NH_4_ levels. This result is consistent with the data obtained in this study (Figs. 5 and 6). The pH could be an alternative parameter for controlling short nitrification for ammonium-rich wastewater.

However, there are other factors that are used to control nitrite oxidation (e.g. SRT, concentration of FNA and temperature). The results presented by Pollice et al. (2002) indicated that at a given temperature (32 °C) and pH (>7.2), the sludge age is a critical parameter for PN when oxygen supply is not limiting (2 mg O_2_/dm^3^). These authors identified that ammonium oxidation to nitrate was successfully obtained for short SRTs (SRT, 10 days). However, very short SRTs could increase the risk of biomass washout.

In the case of FNA, a concentration of 0.22–2.8 mg N-HNO_2_/dm^3^ inhibits the nitrification process (Anthonisen et al. 1976) and 0.02–0.04 mg N-HNO_2_/dm^3^ inhibits only NOB (Zhang et al. 2010). In this study, a very low FNA concentration (less than 0.2 × 10^−5^ N-HNO_2_/dm^3^) did not influence the PN; in contrast, the FA concentration, which varied from 0.83 to 35.86 mg N-NH_3_/dm^3^ (Figs. 5 and 6), had a significant impact.

According to Kim et al. (2006), temperature is another parameter that may influence PN. In addition to the bacterial activity, the temperature also controls the amount of FA and FNA (Eqs. 1 and 2). In the present work, the wastewater temperature was about 20 °C, which is typical for the laboratory-scale tests.

Nitrogen removal via the nitrite pathway offers several advantages over the traditional process, such as the possibility of old landfill leachate treatment using biological methods and reduced operational costs. Additionally, SBR systems may be used as an alternative to expensive leachate treatment technology, such as microfiltration or reverse osmosis. In the studied SBR system, the addition of landfill leachates to wastewater reached 10 % (volume). However, in real systems, this value does not exceed 0.4 %, which facilitates the fulfilment of the standards for treated wastewater. Because leachates are highly variable in quality and quantity, to obtain PN during co-treatment, the boundary conditions should be expressed as ammonia load rather than the optimal volume of the addition of landfill leachates (as it was confirmed by statistical analyses). In this study, the nitrite accumulations occurred when the initial ammonia concentration in the treated mixture exceeded 80 mg N-NH_4_/dm^3^.

## Conclusions

This study suggests that effective co-treatment of municipal wastewater with landfill leachates can be achieved in an SBR. Additionally, PN was achieved for highly ammonia-loaded (above 80 mg N-NH_4_/dm^3^) influent mixtures by reducing the oxygen concentration (1 ± 0.5 mg O_2_/dm^3^) in the presence of FA (above 2 mg N-NH_3_/dm^3^) and by maintaining a pH level above 8 during the nitrification phase. DO was additionally found to be effective in controlling nitrification to nitrite. However, to achieve more efficient nitrogen removal, denitrification should be supplemented by external carbon source when the TN to BOD ratio increases above 0.2 in the treated wastewater and landfill leachate mixture.

Additionally, due to seasonal variability in leachate quality and quantity, a retention tank should be used to provide the stable concentration of ammonia in the influent to WWTP.

However, additional studies are needed to extend these findings to full-scale wastewater treatment plants without risk of nitrification failure. AOB and NOB community composition, activity and diversity appear to be the key factors required for future applications of PN.
